# Planktonic events may cause polymictic-dimictic regime shifts in temperate lakes

**DOI:** 10.1038/srep24361

**Published:** 2016-04-14

**Authors:** Tom Shatwell, Rita Adrian, Georgiy Kirillin

**Affiliations:** 1 Leibniz-Institute of Freshwater Ecology and Inland Fisheries Department of Ecohydrology, Müggelseedamm 310, 12587 Berlin, Germany; 2 Leibniz-Institute of Freshwater Ecology and Inland Fisheries Department of Ecosystem Research, Müggelseedamm 301, 12587 Berlin, Germany; 3 Freie Universität Berlin Department of Biology, Chemistry, Pharmacy, Takustr. 3, 14195 Berlin, Germany

## Abstract

Water transparency affects the thermal structure of lakes, and within certain lake depth ranges, it can determine whether a lake mixes regularly (polymictic regime) or stratifies continuously (dimictic regime) from spring through summer. Phytoplankton biomass can influence transparency but the effect of its seasonal pattern on stratification is unknown. Therefore we analysed long term field data from two lakes of similar depth, transparency and climate but one polymictic and one dimictic, and simulated a conceptual lake with a hydrodynamic model. Transparency in the study lakes was typically low during spring and summer blooms and high in between during the clear water phase (CWP), caused when zooplankton graze the spring bloom. The effect of variability of transparency on thermal structure was stronger at intermediate transparency and stronger during a critical window in spring when the rate of lake warming is highest. Whereas the spring bloom strengthened stratification in spring, the CWP weakened it in summer. The presence or absence of the CWP influenced stratification duration and under some conditions determined the mixing regime. Therefore seasonal plankton dynamics, including biotic interactions that suppress the CWP, can influence lake temperatures, stratification duration, and potentially also the mixing regime.

The mixing regime and thermal structure of lakes have a profound effect on ecosystem functioning because they strongly influence the availability of nutrients, light and oxygen. Dimictic lakes are typically deep and mix only in spring and autumn while stratifying continuously during the warmer months in between^1^. Polymictic lakes are shallow and mix to the bottom intermittently during the heating period. Lakes that are deep enough to stratify for extended periods but shallow enough that they need not stratify continuously over the heating season can potentially be either polymictic or dimictic. We refer to these lakes here as “marginal” because they can be in both mixing classes. Marginal lakes are therefore susceptible to mixing regime shifts, due for instance to climate warming or anthropogenic change^2^,^3^.

Water transparency, or light extinction, determines the depth of penetration of shortwave solar radiation and has a major effect on mixing regime in temperate marginal lakes^2^,^4^. A number of studies based on field data^4^,^5^,^6^,^7^,^8^,^9^,^10^, experimental enclosures^11^,^12^,^13^ and modelling^2^,^14^,^15^,^16^,^17^ have investigated the influence of transparency on the thermal structure of lakes. They unequivocally conclude that a reduction in transparency decreases deep water temperatures, the thickness of the surface (mixed) layer, and the overall heat content of the water body. The increased temperature difference between surface and deep water stabilises thermal stratification. However, the vast majority of these studies deals with deep, seasonally stratified lakes, and relatively little is known about how transparency affects marginal lakes.

Transparency in lakes is determined by the concentration of dissolved organic carbon (DOC), as well as inorganic and organic particulate matter including phytoplankton. Silt can determine transparency in very shallow lakes with high sediment resuspension, or where inflows carry relatively high particulate loads. DOC is typically low in lakes with limestone-rich catchments^18^, and phytoplankton is usually the dominant factor for transparency in the hard water lakes common in Europe. Phytoplankton biomass typically follows a seasonal pattern in temperate meso- to eutrophic lakes^19^,^20^. Following low biomasses in winter, a spring bloom forms from abundant light and nutrients, before collapsing due to nutrient depletion and zooplankton grazing, initiating the CWP. Biomass then increases again forming a summer peak of grazing-resistant species like cyanobacteria and/or an autumn peak typically of diatoms. Although the annual cycle of phytoplankton biomass can be quite variable, the spring bloom and CWP remain to be the most predictable and general seasonal planktonic events^21^. Furthermore, the timing of the spring bloom is sensitive to climate change^22^,^23^,^24^ and the intensity of the CWP is sensitive to trophic interactions between phytoplankton, grazers and fish^25^,^26^,^27^. It is well known that stratification influences phytoplankton blooms^28^,^29^, but the effects of phytoplankton on stratification are still poorly understood. Feedbacks of phytoplankton on thermal structure have been demonstrated in deep lakes^16^ and it has been suggested that phytoplankton bloom timing should also play a role^11^. However, the effect of the distinct seasonal pattern of transparency resulting from phytoplankton biomass on thermal structure and stratification duration, particularly in marginal lakes, has not yet been investigated.

Here we examine the effect of the seasonality of transparency due to phytoplankton, particularly the spring phytoplankton bloom and the CWP, on the thermal structure of small to medium-sized marginal lakes. We used principal component analysis (PCA) to characterise and relate the modes of seasonal variation in transparency to seasonal variation in stratification in the two study lakes in which pelagic chlorophyll concentrations determine Secchi transparency^30^,^31^,^32^. Our hydrodynamic model simulations were performed with an idealized seasonal phytoplankton pattern in a conceptual lake, where the only lake-specific parameters were light extinction coefficient (γ), depth, and wind fetch, thus focusing on general phenomena rather than lake-specific detail. We hypothesize that seasonal phytoplankton dynamics and the CWP affect the stratification duration, thermal structure, and potentially the mixing regime of temperate marginal lakes.

## Results

### Drivers of water transparency

**Figure 1:**
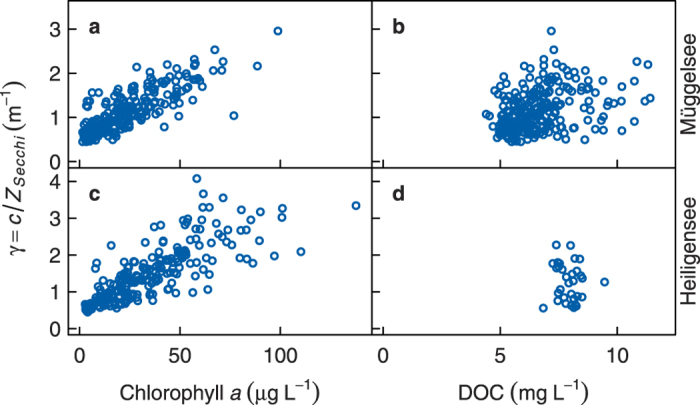
Dependence of extinction (γ) on chlorophyll *a* and dissolved organic carbon (DOC) concentration in Müggelsee (a,b) and Heiligensee (c,d). γ was calculated from Secchi transparency (*Z*_*secchi*_) with equation 3 using *c* = 2.05 for Müggelsee and *c* = 2.13 for Heiligensee (see Methods).

In the two study lakes, Müggelsee (polymictic) and Heiligensee (dimictic), the concentration of chlorophyll *a*, the main light-absorbing material in phytoplankton biomass, was strongly and linearly related to γ or the inverse of Secchi transparency (Müggelsee: *r* = 0.80, *p* < 0.001, *t* = 19.8, *df* = 222, Fig. 1a; Heiligensee: *r* = 0.78, *p* < 0.001, *t* = 18.4, *df* = 214, Fig. 1c). Extinction was also weakly related to DOC in Müggelsee (*r* = 0.35, *p* < 0.001, *t* = 5.8, *df* = 250, Fig. 1b) but not in Heiligensee (*p* = 0.61, *t* = 0.5, *df* = 30, Fig. 1d). The relationships (with 95% C.I.) are given by equation 1 for Müggelsee and equation 2 for Heiligensee:


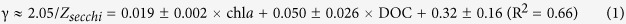



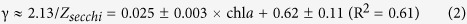


where *Z*_*secchi*_ is the Secchi depth (m), chl*a* is the chlorophyll concentration (μg L^−1^) and DOC is in mg L^−1^. Thus, light absorption through phytoplankton was the main driver of transparency in the study lakes.

### Seasonal variation of transparency and chlorophyll in lake data

**Figure 2:**
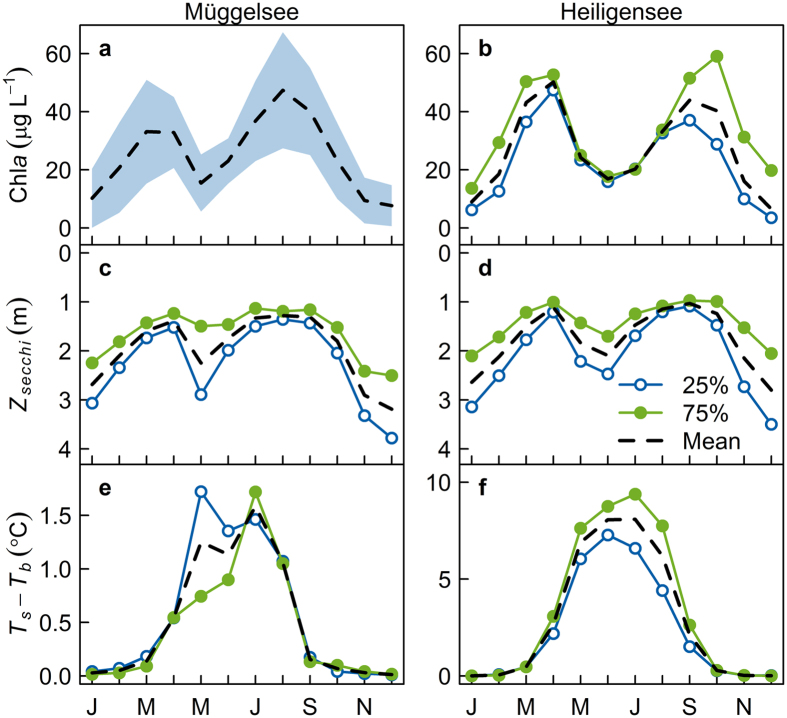
Mean seasonal pattern of chlorophyll *a* (Chl*a*, (a,b)), Secchi transparency (*Z*_*secchi*_, (c,d)) and stratification strength (*T*_*s*_ − *T*_*b*_, (e,f)) in Müggelsee (a,c,e) and Heiligensee (b,d,f). Dashed lines show long term monthly means, lines with symbols show how the first (**b**,**c**,**d**,**f**) or second principal component (**e**) affect the mean seasonal pattern. Open symbols show a low score of the respective principal component (25^th^ percentile in the time series) and closed symbols show a high score (75^th^ percentile). Seasonal patterns represented by like symbols in each lake are correlated. The scores for Chl*a* in Müggelsee are not shown because they were not correlated with *Z*_*secchi*_ or *T*_*s*_ − *T*_*b*_; instead the shaded region in (**a**) represents 1 standard deviation from the mean. Note reversed y-scale for *Z*_*secchi*_ reflecting depth measured downwards.

The long term monthly means of chlorophyll *a* and Secchi transparency in Müggelsee and Heiligensee revealed a distinct bimodal seasonal pattern, which was similar in both lakes (Fig. 2). In winter, chlorophyll was low (<20 μg L^−1^) and transparency was high (>2 m) in both lakes. Subsequently, chlorophyll increased to a maximum in March/April during the spring phytoplankton bloom, which coincided with a minimum in transparency (<1.3 m). Mean chlorophyll then decreased below 20 μg L^−1^ and transparency increased to about 2 m during the CWP in May in Müggelsee or June in Heiligensee. Chlorophyll increased again during the summer phytoplankton bloom in August in Müggelsee and September in Heiligensee, while transparency decreased to a minimum at the same time.

**Figure 3:**
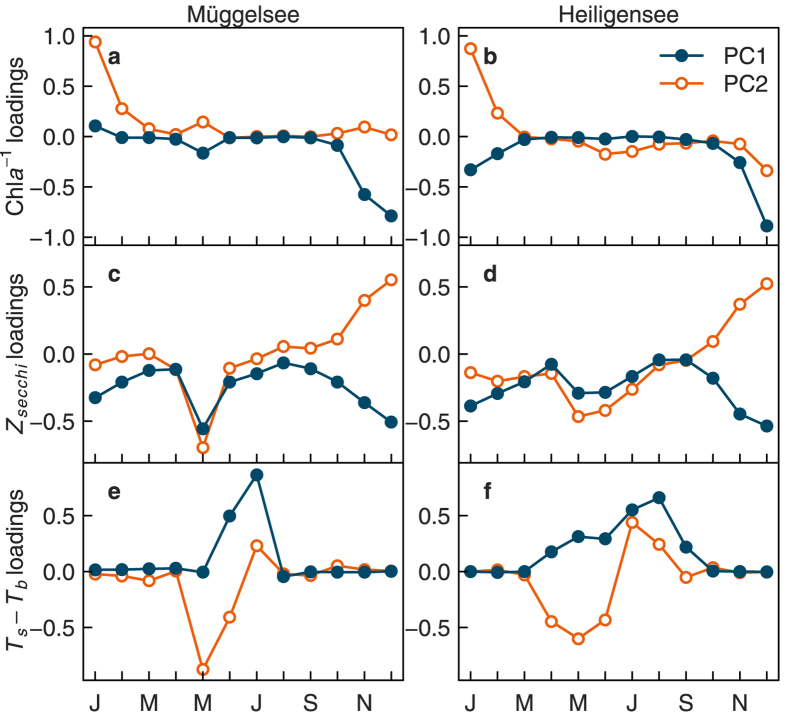
Monthly loadings of the first two components (PC1, PC2) from the principal component analysis of inverse-transformed chlorophyll *a* (Chl*a*^−1^, (a,b)), Secchi transparency (*Z*_*secchi*_, (c,d)) and stratification strength (*T*_*s*_ − *T*_*b*_, (e,f)) in Müggelsee (a,c,e) and Heiligensee (b,d,f).

The PCA revealed similar modes of variation of Secchi transparency in both lakes (Fig. 3c,d). The first principal component (PC1) explained 43% and 49% of the variance of Secchi transparency in Müggelsee and Heiligensee respectively. PC1 had high loadings in spring and summer and low loadings during the CWP in April/May and during winter (Fig. 3c,d), so that PC1 effectively represented the amplitude of seasonal variation. Low PC1 loadings, for instance, corresponded to stronger spring and summer peaks and a more intense CWP, whereas high loadings corresponded to a much weaker CWP and more constant transparency in both lakes (Fig. 2c,d). Since chlorophyll *a* was proportional to the inverse of Secchi transparency (Fig. 1), we performed the PCA on inverse transformed chlorophyll data. Analogous to Secchi transparency, the PCA revealed similar modes of variation in chlorophyll *a* in Müggelsee and Heiligensee (Fig. 3a,b), where PC1 explained 72% and 62% of the variance of chlorophyll *a*, respectively. PC1 tended to represent the size of the spring and summer chlorophyll peaks, where higher scores corresponded to higher peaks.

### Seasonal variation of stratification in lake data

Both lakes were on average isothermal and unstratified from October to March as reflected in near zero values of the mean difference between surface and bottom temperatures (*T*_*s*_ − *T*_*b*_, neglecting inverse stratification in winter and noting that negative values were truncated; Fig. 2e,f). Mean *T*_*s*_ − *T*_*b*_ increased in April and May, peaking in July at 1.6 °C and 8.1 °C in Müggelsee and Heiligensee, respectively.

The modes of seasonality of *T*_*s*_ − *T*_*b*_ in the PCA were also similar in both lakes (Fig. 3e,f), despite the different mixing regimes. PC1 explained 54% and 53% of the variance in *T*_*s*_ − *T*_*b*_ and PC2 explained 18% and 25% of the variance in Müggelsee and Heiligensee respectively. PC1 had highest loadings in June–July in Müggelsee and July-August in Heiligensee and thus primarily represented the variation in summer stratification (Fig. 3e,f). PC2 on the other hand had the highest absolute loading in April in both lakes and mainly represented variation in spring stratification relative to summer.

### Correlation between seasonal chlorophyll, transparency and stratification

In Lake Müggelsee, the yearly scores of PC1 for Secchi transparency were positively correlated with the PC2 scores for *T*_*s*_ − *T*_*b*_ (p = 0.02, *r* = 0.42, *t* = 2.5, *df* = 29). This means that a clearer CWP, and more distinct bimodal pattern of transparency were associated with stronger vertical temperature gradients in spring and weaker gradients in summer (Fig. 2c,e). The scores for chlorophyll were neither correlated with the scores for Secchi depth, nor for *T*_*s*_ − *T*_*b*_ (p > 0.05). In Heiligensee, the PC1 scores for Secchi transparency were significantly positively correlated with the PC1 scores for *T*_*s*_ − *T*_*b*_ (p = 0.02, *r* = 0.49, *t* = 2.4, *df* = 19). Furthermore, the PC1 scores for chlorophyll were significantly correlated with the both the PC1 scores for transparency (p = 0.0006, *r* = 0.71, *t* = 4.2, *df* = 17) and the PC1 scores for *T*_*s*_ − *T*_*b*_ (p = 0.005, *r* = 0.60, *t* = 3.2, *df* = 18). This means that smaller spring and summer chlorophyll maxima were associated with a more intense CWP and stronger bimodal pattern of transparency, which in turn were associated with weaker stratification, especially in summer (Fig. 2b,d,f). We assessed synchrony between the two lakes by comparing the deseasonalised (centred) monthly means of *T*_*s*_ − *T*_*b*_ and Secchi transparency in the two lakes during the period of parallel measurements (1979–2000), considering only the potentially stratified months of April to August. Neither *T*_*s*_ − *T*_*b*_ (p = 0.09, *r* = 0.16, *t* = 1.70, *df* = 108), nor Secchi transparency (p = 0.46, *r* = 0.08, *t* = 0.74, *df* = 93) were significantly correlated in the two lakes. Monthly chlorophyll concentrations did correlate between the lakes however (p = 0.008, *r* = 0.39, *t* = 2.8, *df* = 42), at least during the years when parallel measurements existed (1991–2000). We further checked the principal components for synchrony. PC1 for Secchi transparency was not correlated between the lakes (p = 0.10), nor was PC2 for *T*_*s*_ − *T*_*b*_ in Müggelsee correlated with PC1 for *T*_*s*_ − *T*_*b*_ in Heiligensee (p = 0.55), nor were the PCs for chlorophyll correlated (p = 0.72). Thus transparency and stratification were correlated within each lake, but in a way that was not synchronous between the lakes. In summary, a stronger seasonal amplitude of transparency and a more intense CWP significantly weakened summer stratification and skewed temperature gradients away from summer towards spring in both lakes. This response was independent of whether the lake was polymictic or dimictic.

### Extinction scenarios

**Figure 4:**
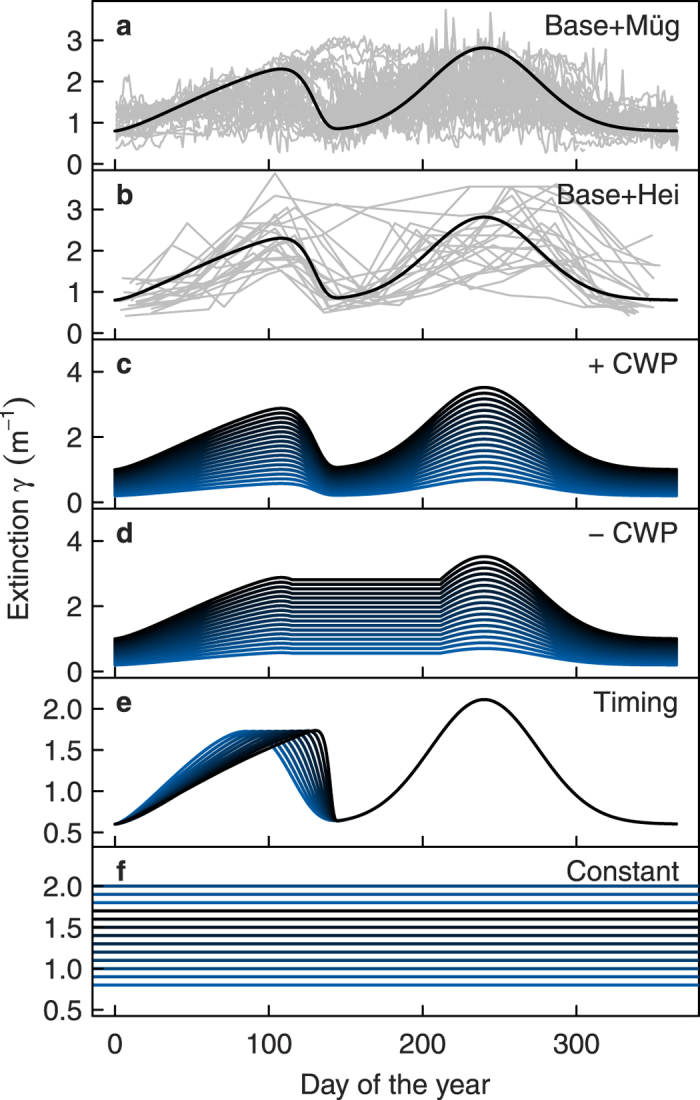
Seasonal extinction scenarios used in model simulations. The base seasonal extinction pattern (black line in (**a**,**b**)) was defined based on long term measured extinction in Müggelsee (grey lines in (**a**), each line representing one year) and Heiligensee (grey lines in (**b**)). Extinction scenarios were then derived from the base extinction pattern by varying annual mean extinction with a clear water phase (CWP, (**c**)) and without a CWP (**d**) or by varying the timing of the spring bloom (**e**). Seasonally variable extinction scenarios were compared with control scenarios of constant extinction (**f**).

Apart from extinction being overall slightly higher in Heiligensee than Müggelsee, the seasonal extinction characteristics in the two lakes were very similar (Table 1). Both lakes exhibited a CWP in most but not all years. Absence of the CWP was evident from high extinction extending from spring through summer in individual years (Fig. 4a,b). Based on these characteristics and the Plankton Ecology Group model^19^,^20^, we defined a bimodal base seasonal extinction pattern, which described the mean seasonal extinction dynamics in Müggelsee and Heiligensee well (Fig. 4a,b). The extinction scenarios reflected the known modes of variation in the seasonal extinction pattern, including variation in the annual mean extinction (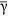), achieved by scaling the base extinction pattern slightly beyond the ranges observed in Müggelsee and Heiligensee (Fig. 4c), suppression of the CWP according to de Senerpont Domis, *et al.*^27^ (Fig. 4d), and variation in timing of the spring bloom, covering the long term range observed in Lake Müggelsee^23^ (Fig. 4e). In simulations, these scenarios were compared with control scenarios of constant extinction at the same 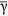 (Fig. 4f).

### Model validation

**Figure 5:**
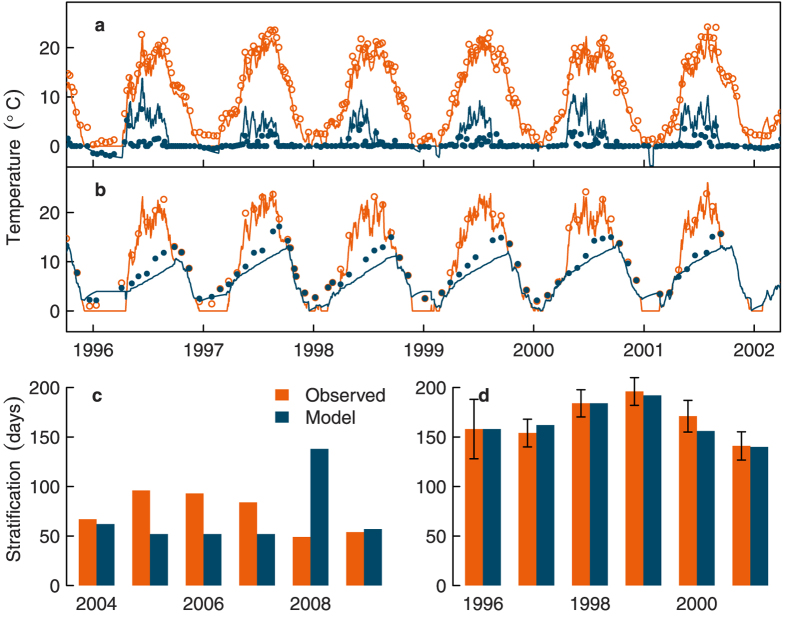
Model validation against measured temperatures and stratification in Müggelsee (a,c) and Heiligensee (b,d). In the upper panels, open orange symbols show surface temperatures, closed blue symbols show surface-bottom temperature differences (**a**) or bottom temperatures (**b**). Lines show the corresponding simulated temperatures. In the lower panels, vertical bars show the observed (orange) and modelled (blue) total number of stratified days per year. Error bars in (**d**) were estimated from the sampling interval. We used the period 2004–2009 in (**c**) for Müggelsee because high frequency temperature profiles were available only after 2004. The model was parameterised for a conceptual lake similar to both Müggelsee and Heiligensee. It was run with the same meteorological forcing for both lakes with the exception of a 50% increase in wind speed for Müggelsee.

Using the base seasonal extinction pattern in the hydrodynamic model, we simulated *T*_*s*_ and *T*_*b*_ in the conceptual marginal lake, which was designed to be similar morphologically to Müggelsee and Heiligensee (Table 1). Comparing the simulated temperatures in the conceptual lake with measured temperatures in Müggelsee and Heiligensee (the only difference being that wind speed was increased by 50% for comparisons with Müggelsee to reflect its more wind exposed location than Heiligensee) showed good agreement (Fig. 5a,b). The root mean square error for *T*_*s*_ and *T*_*b*_ in Müggelsee was 1.8 °C and 2.8 °C (n = 1185) respectively, and in Heiligensee was 2.1 °C and 2.7 °C (n = 382) respectively. The model successfully portrayed the dimictic regime of Heiligensee and the polymictic regime of Müggelsee. The model estimated the stratification duration in Heiligensee relatively well (Fig. 5c,d; model mean ± s.d.: 147 ± 47 days, observed: 168 ± 17 days) as well as timing of onset (model: day 112 ± 16, observed: day 96 ± 16) and end (model: day 254 ± 40, observed: day 264 ± 13). The model did not necessarily capture all the individual stratification events in Müggelsee, but it did describe the timing of earliest stratification (model: day 97 ± 8, observed: day 82 ± 12) and latest stratification (model: day 237 ± 24, observed: day 263 ± 16) and also the total number of stratified days per year well (model: 58 ± 26 days, observed: 73 ± 14 days).

### Effect of constant extinction on thermal properties

**Figure 6:**
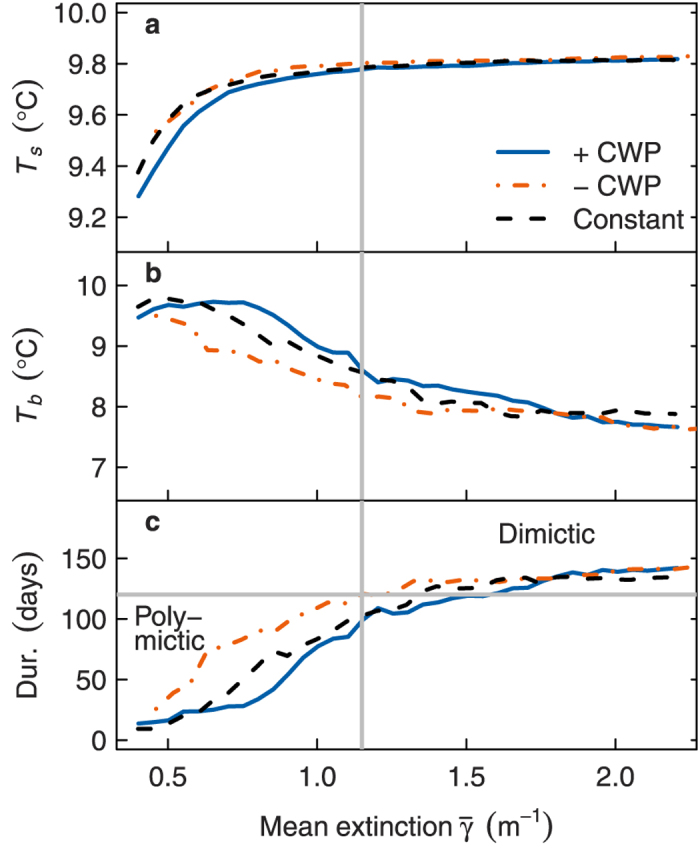
Simulated effect of annual mean extinction,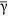, on mean lake thermal properties under constant extinction, or variable extinction scenarios with and without clear water phase (CWP). *T*_*s*_: surface temperature, *T*_*b*_: bottom temperature, Dur.: stratification duration. Grey dashed lines denote a transitional region between polymixis and dimixis at Dur = 120 days. The lake becomes dimictic at higher extinction when the CWP is present than when it is absent.

Model simulations with the constant extinction (control) scenario demonstrated the nonlinear effects of extinction on lake thermal properties. The conceptual lake was polymictic (longest stratified period <120 days) below 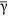 ≈ 1.3 m^−1^ and dimictic above this threshold based on means of the whole simulation period of 1980–2010 (Fig. 6c). At 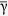 < 0.5 m^−1^ (polymictic regime), extinction affected both *T*_*s*_ and *T*_*b*_ because the lake was generally well mixed (Fig. 6a,b). At 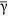 > 1.5 m^−1^ (dimictic regime) the effect of extinction on lake temperature and thermal structure diminished, with stratification duration and bottom temperatures relatively stable at about 150 days and 8 °C, respectively (Fig. 6). However, there was a transitional region between stable polymixis and stable dimixis at intermediate extinction (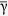 = 0.5–1.5 m^−1^), where increasing extinction decreased *T*_*b*_ (by 2.5 °C) and increased stratification duration (by 128 days).

### Effect of seasonally variable extinction

Seasonally variable extinction produced striking effects on thermal structure in the transitional range of 0.5–1.5 m^−1^. Variable extinction with a CWP weakened thermal gradients and stratification compared to constant extinction within the range 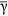 = 0.6–1.0 m^−1^, as evident from higher *T*_*b*_ and shorter stratification (Fig. 6). On the other hand, in scenarios without a CWP, temperature gradients and stratification duration were considerably higher at the same 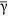 across the whole transitional range (0.5–1.5 m^−1^). For example, at 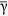 = 0.7 m^−1^ mean *T*_*b*_ was 1 °C lower and stratification duration was on average 49 days longer when the CWP was absent than when it was present. Accordingly, at 1.0 < 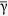 < 1.3 m^−1^, the presence or absence of the CWP determined whether the lake was dimictic or polymictic.

**Figure 7:**
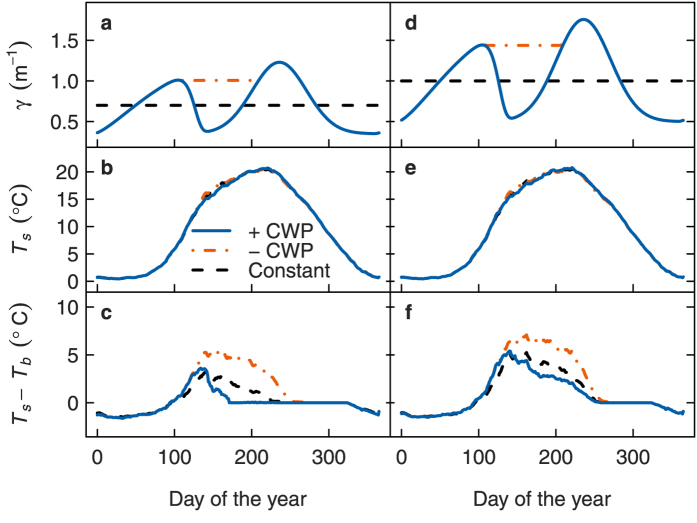
Seasonal development of mean simulated lake thermal characteristics, comparing constant extinction (dashed lines) and seasonally variable extinction scenarios with a clear water phase (CWP, solid lines,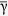 = 0.7 m^−1^ (a,b,c) or 1.0 m^−1^ (d,e,f)) and without a CWP (dot-dashed lines, 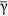 = 0.8 m^−1^ (a,b,c) or 1.1 m^−1^ (d,e,f)). γ: extinction, *T*_*s*_: surface temperature, *T*_*b*_: bottom temperature.

The reasons become clear when considering the annual cycle of lake temperature. With seasonally variable extinction under relatively clear conditions (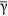 = 0.7 m^−1^), the spring bloom initially increased temperature gradients in spring compared to constant extinction, but the subsequent CWP broke down these gradients which then remained low throughout summer (Fig. 7c). This is similar to the response observed in Müggelsee where a more distinct bimodal pattern of transparency with a stronger CWP increased *T*_*s*_ − *T*_*b*_ in spring but decreased it in summer (cf. Fig. 2e). When the CWP was absent however, high temperature gradients persisted from spring till the end of summer, strongly increasing stratification duration (Fig. 7c). Under higher overall extinction (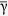 = 1.0 m^−1^), the presence of the CWP could not completely break down temperature gradients, but still substantially decreased *T*_*s*_ − *T*_*b*_ throughout summer, leading to earlier onset of mixing and shorter stratification (Fig. 7f). This is similar to the response observed in the more stably stratified Heiligensee with similar *T*_*s*_ − *T*_*b*_, where a more distinct bimodal pattern of transparency decreased *T*_*s*_ − *T*_*b*_ in summer relative to spring (cf. Fig. 2f). Overall the effect of seasonal variability of extinction on surface temperature was small at 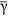 > 0.7 m^−1^, causing only a slight decrease during the CWP (Fig. 7b,e), which cancelled out in long term means (Fig. 6a).

### Bloom phenology and sensitivity to timing

Scenarios with variable spring bloom timing showed that a delay in spring bloom timing from day 90 to day 120 (approximate range observed in Lake Müggelsee), increased thermal stability of the water column slightly as evident in higher *T*_*s*_ − *T*_*b*_ beginning near the spring bloom, and persisting as long as *T*_*s*_ − *T*_*b*_ was positive. Bloom timing had little effect on *T*_*s*_ or overall stratification duration.

**Figure 8:**
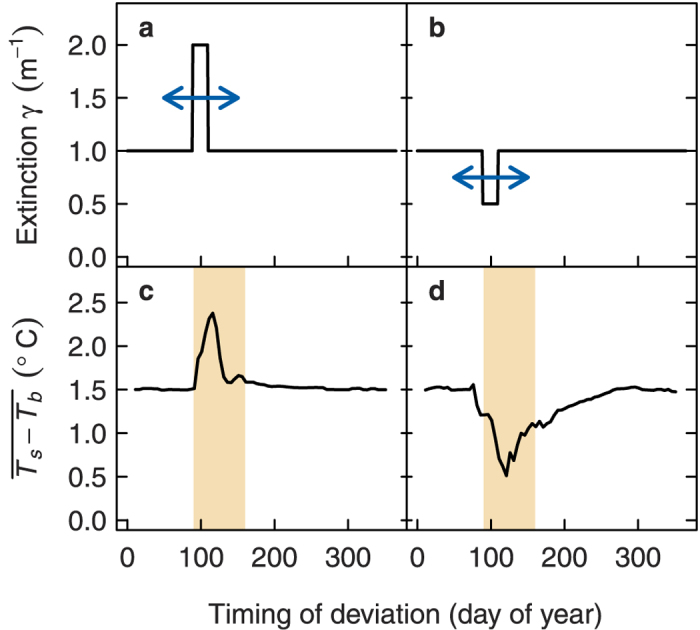
Effect of a short term deviation in extinction at different times of year on the annual mean difference between surface (*T*_*s*_) and bottom temperature (*T*_*b*_) (c,d). The deviation was created by doubling (**a**) or halving (**b**) a constant arbitrary baseline extinction (1.0 m^−1^) for 20 days. Simulations were performed with the deviation midpoint shifted to different days of the year.

Since the CWP and the spring bloom produced larger responses in stratification than the summer bloom, we performed a sensitivity analysis to determine whether extinction affected thermal structure more at certain times of the year than others. Here we considered a scenario with a constant, arbitrary baseline extinction of 1.0 m^−1^ except for a brief deviation, where we either doubled (Fig. 8a) or halved (Fig. 8b) the extinction for a duration of 20 days. We then performed a series of simulations each time shifting this deviation to a different time of year to test the sensitivity of timing on stratification. A short term increase or decrease in extinction had a stronger effect on vertical temperature gradients during a critical window during spring from day 90 to day 160 (April–June, Fig. 8c,d). The greatest effect on temperature gradients however was clearly around day 110 to 120, when for instance doubling the extinction for 20 days increased annual averaged vertical temperature differences from 1.5 °C to 2.5 °C. Outside this critical window, a short term increase in extinction had a much smaller effect, with mean vertical temperature differences stable at 1.5 °C.

### Mixing regime shift sensitivity

**Figure 9:**
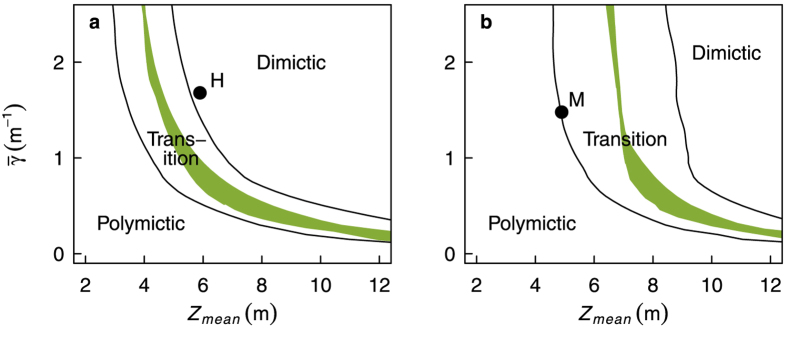
Sensitivity of mixing regime to mean extinction (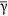), mean lake depth (*Z*_*mean*_), and the clear water phase (CWP) for a lake with 1000 m fetch similar to Heiligensee (a), and for a lake with 4000 m fetch and increased wind speed by 50%, similar to Müggelsee (b). The shaded area represents the region that is on average only polymictic when the CWP is present. Lines show 10^th^ and 90^th^ percentiles, delineating the transitional conditions under which lakes shifted between polymictic and dimictic regimes in at least 10% of simulated years. The circles marked “M” and “H” show the average observed conditions in Müggelsee and Heiligensee, respectively.

We assessed the sensitivity of the mixing regime to extinction and presence or absence of the CWP at different depth and fetch combinations. At high mean depth (ca. 10 m), lakes were only polymictic when they were very clear, and a relatively small increase in extinction was sufficient to shift the regime to dimictic (Fig. 9). Shallower lakes shifted from polymictic to dimictic at higher extinctions. With increasing extinction, the critical depth at which the lake shifted regimes tended asymptotically towards a specific depth, which was always polymictic regardless of extinction. This depth was about 3 m (Fig. 9a) or 6 m (Fig. 9b) for the wind speed/fetch configurations of Heiligensee and Müggelsee, respectively, and was therefore lake-specific. The region where the presence or absence of the CWP determined the mixing regime was broader at intermediate depth (5–8 m) and extinction (0.5–1.5 m^−1^) and became narrow at more extreme combinations. The range of extinctions in which the CWP on average determined the mixing regime was lake-specific and shifted with depth, but spanned about 0.3 m^−1^ at any one fixed depth between 5 and 8 m. The CWP played a negligible role in determining the mixing regime of marginal lakes that were very shallow and turbid. The analyses suggest that Müggelsee is unlikely to become dimictic through a change in transparency, whereas Heiligensee could potentially become polymictic were it to become very clear with a strong CWP.

## Discussion

We demonstrated that stratification duration and the mixing regime of marginal lakes may respond strongly to seasonal changes in phytoplankton biomass. Our study showed that cardinal planktonic events in spring, especially the CWP, potentially have a large influence because they fall within a critical window during which transparency has a much stronger effect than at other times of the year. The model simulations indicated the existence of certain depth and extinction combinations where the presence or absence of the CWP altered the average mixing regime. The empirical results demonstrated that a stronger seasonal pattern associated with a more intense CWP decreased summer stratification relative to spring. We therefore confirm our hypothesis that seasonal phytoplankton dynamics and the CWP significantly affect the stratification duration, thermal structure and mixing regime of temperate marginal lakes.

Several recent studies have investigated the transparency-mediated effects of phytoplankton on the thermal conditions in lakes, but none have considered the known seasonal pattern of biomass resulting from plankton succession on stratification duration and the mixing regime, and few have specifically considered shallow and/or turbid lakes. Studies of deep clear lakes showed that there is an interaction between phytoplankton and stratification that influences thermal structure^16^ but suggested that intra-annual variations in extinction may be small and thus have minor importance relative to interannual variation^15^. However, the seasonal transparency pattern in deep oligotrophic lakes differs from that in shallow, eutrophic lakes^19^. Furthermore, transparency can have a very large effect on stratification duration in temperate polymictic^2^,^33^,^34^ but also subtropical monomictic lakes^9^, which does not seem to be the case in deep dimictic temperate lakes. Our results are in line with these conclusions and suggest that the effect of transparency is a lot stronger in marginal lakes because its seasonal variability has a greater effect in the range between stable polymixis and stable dimixis. Thus we conclude that not only the annual mean transparency, but also the seasonal variation in transparency and the CWP play an important role, particularly in marginal lakes.

Our results are in line with previous studies which show that increasing annual mean extinction decreases the deep water temperature^11^,^14^. The novelty of our results lies in the specific response of marginal lakes to variations in mean extinction. In very clear marginal lakes, variations in mean extinction strongly affect the mean lake temperature, which is also the surface temperature because stratification is rare^4^. Very turbid marginal lakes respond to a change in transparency similarly to deeper lakes, because both are dimictic in temperate zones^2^. However, the strongest effect of extinction on stratification was observed in lakes with intermediate transparency (0.5 < 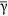 < 1.5 m^−1^) because they are on the transition between polymixis and dimixis, and therefore most susceptible to mixing regime shifts mediated by transparency. The sensitivity analysis suggested that at these intermediate transparencies and the climatic forcing we used, lakes between about 6 and 9 m deep are susceptible to switch predominant mixing regimes. Furthermore, accounting for climatic variability and the broader range of extinction encountered in most lakes (say 0.3–2.5 m^−1^), temperate lakes about 4–10 m deep can potentially be either polymictic or stratified, depending on transparency. This agrees very closely with empirical observations: Canadian Shield lakes shallower than 5 m were generally polymictic and those deeper than 10 m were generally stratified, whereas the mixing regime of the lakes between these depths depended on transparency^4^. The sensitivity analysis further implied that doubling the extinction (or halving the Secchi transparency) in a particular lake within these depth ranges could be sufficient to shift the mixing regime from stably polymictic to stably dimictic. This agrees with an observed shift from polymixis to dimixis in a small clear marginal lake (8.1 m deep) when light extinction increased from 0.25 to 0.44 m^−1^ following several years of increased precipitation^34^.

Our approach of simple one-dimensional modelling combined with multivariate statistical analysis was designed to isolate the effect of seasonal change in transparency on mixing, which is usually masked by lake morphology or synoptic weather conditions in multiple lake comparisons or long term field data. The interactions between thermal structure and phytoplankton growth in lakes are complex, involving factors like light, nutrients and other trophic levels^35^,^36^. Stratification and transparency also vary spatially due to lake morphology^37^ or the vertical distribution of phytoplankton^38^. For example, surface blooms of cyanobacteria have been observed to increase surface water temperature and water column stability^39^,^40^,^41^,^42^. Irregular morphology may cause some lake zones to stratify while others are mixed. We assumed the conceptual lake was on average “mixed” when it was isothermal down to the mean lake depth, ignoring potential localised stratification at deeper points. This simplification could cause problems when applying the model to reproduce an exact 3-d density distribution in lakes with very irregular morphometry. This is however not the case for both lakes under study, which have regular morphometry and weak vertical volume development^2^. While more complex coupled models with feedbacks between phytoplankton and thermal structure^16^ and three dimensional models^37^ can better account for this additional variability, such models must consider more lake-specific factors like inflows, nutrient inputs, complex morphology and food webs. Furthermore, feedbacks can make it very difficult to isolate the causal effects of transparency on thermal structure. To maximize the generality of the results, we focused on averaged responses using a simple uncoupled model with transparency as an independent variable, and only fetch and depth as lake-specific parameters. Furthermore, cardinal planktonic events like the spring bloom and CWP, including the overall seasonal pattern of phytoplankton biomass, are common and well established phenomena in temperate eutrophic lakes^19^,^27^. Our model generally reproduced the observed thermal characteristics in Lakes Müggelsee and Heiligensee. Heiligensee is more wind-sheltered than Müggelsee, which can substantially influence mixing, so we adjusted the wind speed to reflect this in the model validation. This adjustment was an attempt to reduce the potential influence of a factor of unknown magnitude, and although realistic, was somewhat arbitrary in the absence of direct measurements. Increasing the wind speed for instance decreases stratification duration and increases the extinction at which lakes switch between polymictic and dimictic regimes, as can be seen by comparing Fig. 9a,b. The bottom temperatures in the modelled conceptual lake were slightly lower than the observed bottom temperatures in Heiligensee, which in part is because the model parameters were not exactly tuned for either Heiligensee or Müggelsee. Nevertheless the errors for *T*_*b*_ were not abnormally high compared to other models, which do not reproduce temperatures at intermediate depths as accurately as at the surface or at high depths^43^,^44^,^45^,^46^,^47^ (see Supplementary Discussion for a comparison of model errors). This is not surprising for marginal lakes because by definition, the mean lake depth is similar to the thermocline depth, where temperatures gradients can be high.

We analysed two lakes of very similar depth, climate and transparency. The reason the lakes nevertheless had different mixing regimes may be attributed to the slightly larger mean depth (5.9 vs 4.9 m) and the shorter fetch (1000 vs. 4000 m) of Heiligensee, resulting in the aspect ratio difference of 0.006 vs. 0.001 (see Spigel and Imberger^48^ on the aspect ratio effects on the mixing regime). Interestingly, stratification in both lakes responded similarly to transparency regardless of the mixing regime: stronger seasonal variation of transparency and a clearer CWP was related to weaker summer stratification, and in the case of Müggelsee, stronger spring stratification. Phytoplankton chlorophyll was clearly driving transparency in both lakes as demonstrated by the strong correlations. While PC1 for chlorophyll correlated with the PC1s for transparency and stratification in Heiligensee, it did not in Müggelsee, which may have been due to the substantially shorter chlorophyll time series in this lake. However, correlation alone does not prove that transparency caused the change in stratification because stratification also influences phytoplankton growth. For instance, longer stratification events were shown to influence phytoplankton and promote cyanobacterial dominance in Müggelsee^49^, so there is most likely an interaction between stratification and bloom formation. This interaction makes it difficult to infer causality from field data, so we used the dynamic model, in which causality is explicit, to help interpret whether phytoplankton-mediated changes in transparency also alter stratification. The model simulations of the conceptual lake indicated that a more intense CWP compared to constant extinction weakens summer stratification relative to spring under both weak stratification (summer *T*_*s*_ − *T*_*b*_ ~ 0–5 °C) and stronger stratification (summer *T*_*s*_ − *T*_*b*_ ~ 5–10 °C) as observed in Müggelsee and Heiligensee. Furthermore, we did not detect any significant synchrony in the correlated principal components between the two lakes, suggesting that internal dynamics might have a stronger influence than regional climate, which should affect both lakes similarly. Taken together we therefore conclude that, in addition to the well-known effects of stratification on bloom formation, seasonal variability of transparency through phytoplankton also significantly influences stratification in marginal lakes regardless of whether predominantly polymictic or seasonally stratified.

One of the major findings of our study was the importance of the CWP for the thermal structure and mixing regime. Although CWPs are common in temperate lakes^27^, their effect on stratification has not been investigated previously. Our finding that a change in extinction has the strongest effect in spring when the rate of warming is highest agrees with another study, which concluded that light extinction dynamics only influenced the lake thermal structure during heating phases^16^. In fact, the mean observed timing of the CWP and spring bloom in Lake Müggelsee^23^ coincided exactly with the time of maximum effect of extinction predicted in the sensitivity analysis (days 110–120), which explains why the CWP and spring bloom have such a strong effect on thermal structure. This was clearly evident in the statistical analysis of Müggelsee and Heiligensee, where a smaller spring bloom and clearer CWP weakened summer stratification relative to spring. The simulations with the conceptual lake reproduced this stratification response to seasonal transparency, albeit at somewhat lower 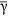, delivering an explanation for this behaviour. Whereas the spring bloom initially strengthens stratification, under thermally stratified conditions, the CWP reduces vertical temperature differences by allowing radiation to penetrate to and heat deeper water layers, and thus potentially alter the mixing regime.

The importance of the CWP and the weather conditions during the spring critical window also explains the anomaly in the model validation against the stratification duration observed in Müggelsee in 2008 (Fig. 5c). Comparing for instance May of each year, 2008 had the highest mean global radiation, the third highest mean air temperature, and the lowest mean wind speed in the 30 year data period. Accordingly, using a standard extinction scenario in the validation, the model predicted strong stratification in Müggelsee beginning in late April, which was stable enough to persist into summer. Whereas this led to a long period of stratification in the simulation, the observed stratification duration in Müggelsee was considerably shorter. In the lake, strong stratification also developed in late April but had disappeared again by mid June. The reason is likely that the CWP in May 2008 (mean Secchi depth 4.1 m) was one of the most intense in the 35 year record, being only exceeded in May 1998 (mean Secchi depth 4.5 m).

In our simulations, the conceptual lake was dimictic without a CWP but polymictic with a CWP at 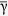 = 1.0–1.3 m^−1^. This range is lake-specific as illustrated by the sensitivity analysis and the different mixing regimes of Müggelsee and Heiligensee at similar transparency and depth. The CWP was more important at intermediate extinction and depth ranges than under extremely clear or extremely turbid conditions, under which the CWP generally does not occur or is not a prominent feature of plankton seasonality^19^,^27^. Apart from lake depth, the intensity of the CWP should play an important role because a change in extinction has a greater effect when extinction is low^11^,^15^,^16^. The CWP can be suppressed by high fish predation of zooplankton^26^, or trophic mismatch^25^,^27^. Experimental studies in shallow to medium depth eutrophic systems showed that phytoplankton and its interactions with fish and zooplankton influence thermal structure and mean lake temperature^11^,^12^,^13^ but our results suggest that such interactions affect stratification duration and thus potentially also the mixing regime.

Kirillin^2^ simulated climate change effects on mixing in temperate lakes of varying depth (1–100 m), and predicted that many polymictic lakes should become dimictic during the first half of the 21^st^ century as stratification events extend to span the whole summer. Since this shift in polymictic lakes is very sensitive to light extinction, biotic interactions are likely to play a role. Research suggests that warming should decrease transparency through higher phytoplankton biomass^50^ or higher dissolved organic carbon concentrations^51^,^52^. Warming and eutrophication may also decrease the occurrence of the CWP^27^. Therefore biotic interactions may accelerate the mixing regime shifts in temperate marginal lakes expected due to climate change.

## Methods

### Study sites and field data

Lake Müggelsee^30^ is a shallow, polymictic, eutrophic lake (maximum depth 8 m, surface area 7.3 km^2^, 52.44° N, 13.65° E, other details in Table 1). Lake Heiligensee^32^ is a shallow, dimictic, eutrophic lake (maximum depth 9.5 m, surface area 0.3 km^2^, 52.605° N, 13.216° E). Both lakes are located in Berlin, Germany. Water temperature profiles were measured at 0.5 m depth increments in Müggelsee at weekly intervals from 1979 to 2013 between 9 and 10 a.m. (and thus largely excluded diurnal stratification events). Hourly profiles at a different sampling site in Müggelsee were available from 2004 on and were only used to calculate the total number of stratified days per year. Temperature profiles in Heiligensee were measured at 1.0 m increments at biweekly to monthly intervals from 1975 to 2001. Secchi depth in both lakes was measured using a standard 20 cm Whipple disk. Underwater light was measured simultaneously using two spherical photosynthetically available radiation (PAR) sensors (LI-193SA, LICOR, Nebraska) vertically separated by 0.5 m. In Müggelsee the sensors were located at 0.75 and 1.25 m depth, measuring PAR continuously from 1993–2013. In Heiligensee PAR was measured at 0.2/0.7 m, 1.0/1.5 m and 1.5/2.0 m at monthly intervals from 1996–2000. In Lake Müggelsee, chlorophyll *a* was available from 1991 on and was measured by High Performance Liquid Chromatography according to Shatwell, *et al.*^36^. In Lake Heiligensee chlorophyll *a* was measured photometrically after hot ethanol extraction^32^ from 1978 to 2001. DOC was measured by non-dispersive infrared sensing after combustion with an N/C analyser (Shimadzu in Heiligensee, or a Jena Analytics multi N/C 2100 in Müggelsee).

### Data preparation and calculations

The scalar light extinction coefficient, γ, was calculated from the simultaneous PAR measurements (excluding measurements before 10 a.m. and after 2 p.m.) according to the Lambert-Beer law. Based on parallel measurements, γ was related to Secchi transparency (*Z*_*secchi*_) by equation (3) after Poole and Atkins^53^:


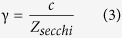


with the dimensionless constant *c* = 2.05 ± 0.07 (95% C.I.) in Müggelsee (n = 300) and *c* = 2.13 ± 0.20 in Heiligensee (n = 52). In years when the spherical sensors were not available in Lake Müggelsee (1979–1992), γ was estimated from the Secchi depth and from photometric extinction measurements as described in Nicklisch *et al.*^54^. In Heiligensee, γ was reconstructed for 1975–2001 from the longer Secchi depth time series.

In analyses using field data, *T*_*s*_ was estimated as the mean temperature in the 0–1 m depth layer and *T*_*b*_ as mean temperature in the 5–6 m layer, reflecting the mean depths of the two lakes. Monthly means of Secchi depth, chlorophyll *a*, and *T*_*s*_ − *T*_*b*_ were calculated by first interpolating these variables linearly over time at daily increments, and then averaging the daily values over the months. Negative values of *T*_*s*_ − *T*_*b*_ were truncated because winter stratification was not of direct interest in the analysis. In field data and model output, stratification was inferred when *T*_*s*_ − *T*_*b*_ exceeded a threshold of 0.5 °C, which has been demonstrated to restrict lake mixing and initiate oxygen depletion^55^. Due to the self-similar profile used in FLake, the model theoretically indicates stratification when *T*_*s*_ − *T*_*b*_ is as low as 0.1 °C. Our threshold of 0.5 °C is lower than the 1 °C threshold used in some other studies^33^,^56^, but was chosen as a compromise reflecting model sensitivity and the relatively small temperature gradients potentially encountered in polymictic lakes. We classified the lakes to be dimictic when the longest uninterrupted period of stratification each year exceeded 120 days and polymictic otherwise^2^.

### Model and external forcing data

Lake temperature and thermal regime of a conceptual lake similar to Müggelsee and Heiligensee were calculated with the lake temperature and mixing model FLake^2^,^57^,^58^. FLake is a one-dimensional model that is described in detail in Kirillin^2^. In short, the model is based on a two-layer parametric representation of the vertical temperature structure with a vertically homogeneous upper layer and a lower, stably-stratified layer, parameterized using a polynomial self-similar representation of the temperature profile. The depth of the mixed layer is computed from the prognostic entrainment equation in convective conditions, and from the diagnostic equilibrium boundary-layer depth formulation in conditions of wind mixing against the stabilizing surface buoyancy flux. Surface heat and momentum fluxes are calculated with the Mironov, *et al.*^59^ algorithm, and short-wave solar radiation based on exponential decay. The model includes modules for ice^60^ and heat storage by the lake sediment^61^. FLake indirectly assumes the lake to have a constant cross-sectional area and flat bottom (maximum depth is equal to the mean depth). Hence, the bottom temperature in FLake is the temperature in a hypothetical lake of regular form with the same area and volume as the real lake. The result of this simplification is the possibility to apply the self-similarity approach to the temperature profile and an enormous increase of computational speed (FLake is at least 10^2^ times faster than any existing one-dimensional lake model). The side effect is a divergence of the simulated near-bottom temperatures from the observed ones. The inconsistencies in the bottom temperature are however comparable to those found in other lake models, and can be reduced by fitting the model to a certain lake. In this study we intentionally avoid any fitting, using the model as a process-based mechanistic representation of heat transport in an ‘ideal lake’ capturing the general dynamics and mean behaviour observed in two real lakes.

Model simulations with FLake were forced using meteorological data (3-hourly resolution) from 1980–2010 from a weather station in Potsdam, Germany, close to Lakes Müggelsee and Heiligensee. Some missing cloud cover data and other small data gaps were filled with data from nearby stations. The model forcing variables include solar radiation, air temperature, wind speed, humidity, and long wave radiation estimated from cloud cover.

### Extinction scenarios

Seasonally variable extinction scenarios assumed a bimodal peak in γ, with the first peak corresponding to the spring phytoplankton bloom and the second peak corresponding to the summer phytoplankton maximum. The extinction during the spring phytoplankton bloom was described by a Weibull function, *w*(*t*), (equation 4) according to Rolinski, *et al.*^62^:


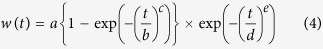


where *t* is the independent variable (time, day of the year), and *a, b, c*, *d*, and *e* are free parameters. The extinction during the summer phytoplankton maximum was described by a Gauss (bell) function, *g*(*t*) (equation 5):


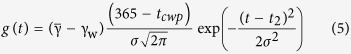


where 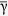 is the annual mean extinction, γ_w_ is the background extinction coefficient of water plus dissolved and (non-algal) particulate light absorbing matter (m^−1^), *t*_*cwp*_ is the timing of the clear water phase, *t*_2_ is the timing of the summer maximum, and *σ* is the standard deviation of the curve (all in day of the year). The overall seasonality of extinction is then given as the sum of equation 4, equation 5, and the background extinction:


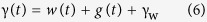


Equations (4) were parameterized with the values in Table 1 in addition to *σ* = 35, *a* = 2.39, *b* = 102.53, *c* = 1.39, *d* = 131.2, *e* = 16.41.

### Statistical analyses

For the PCA, the monthly means of *T*_*s*_ − *T*_*b*_, Secchi depth, and chlorophyll *a* were each compiled into a matrix with one row per year and 12 columns for the months of the year, and then the PCA was performed with the year as the dependent variable and the months as the independent variables^63^. Data were centred but not scaled to maintain comparability of the seasonal pattern. Different modes of seasonality were inferred from the loadings of each month in the respective principal components (PCs). The scores of the PCs for *T*_*s*_ − *T*_*b*_ were compared with the scores of the PCs for Secchi depth and chlorophyll *a* using Pearson correlations. Plots of residuals versus fitted values, Cook’s distances and quantile-quantile plots were inspected to assess randomness of residuals, homogeneity of variance and normality. There was no evidence of non-normality or heteroscedasticity in the data used for statistical comparisons. All tests used a two-tailed alpha level of 0.05 and all statistical analyses were performed with R version 3.1.3^64^.

## Additional Information

**How to cite this article**: Shatwell, T. *et al.* Planktonic events may cause polymictic-dimictic regime shifts in temperate lakes. *Sci. Rep.* **6**, 24361; doi: 10.1038/srep24361 (2016).

## Supplementary Material

Supplementary Information

## Figures and Tables

**Table 1:** Seasonal extinction characteristics in Müggelsee, Heiligensee, and the conceptual lake used in model simulations.

	Müggelsee	Heiligensee	Conceptual lake
Mean depth (m)	4.9	5.9	5.5
Typical fetch (m)	4000	1000	2000
Annual mean Secchi transparency (m)	2.0 ± 0.3	1.8 ± 0.4	–
Annual mean extinction ( 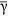 , m^−1^)	1.46 ± 0.29	1.68 ± 0.48	1.6 (0.4–2.2)
Mean peak extinction in spring (γ_1_, m^−1^)	2.2 ± 0.48	2.6 ± 0.92	2.3 (0.6–3.2)
Mean peak extinction in summer (γ_2_, m^−1^)	1.98 ± 0.41	2.83 ± 0.91	2.8 (0.7–3.9)
Mean background extinction (γ_w_, m^−1^)	0.78 ± 0.25	0.92 ± 0.45	0.8 (0.2–1.1)
Mean timing of CWP (t_cwp_, day of year)	143 ± 11^23^	146 ± 14	144 (–)
Mean timing of spring peak, (t_1_, day of year)	109 ± 20^23^	107 ± 12^a^	108 (85–130)
Mean timing of summer peak, t_2_ (day of year)	ca. 230	ca. 250	240 (–)

Means ± s.d. are given for Müggelsee and Heiligensee, and parameter values for the base seasonal extinction pattern are given for the conceptual lake (equations 4–6 in Methods), along with the range used in simulations (in parentheses).

^a^In Heiligensee, the mean timing of minimum transparency was used, which differed slightly from the timing of peak spring biomass (day 91).
