# Supplementary material for: Planktonic events may cause polymictic-dimictic regime shifts in temperate lakes
Source: Sci Rep. 2016 Apr 14;6:24361. doi: 10.1038/srep24361 (PMC4830945; doi:10.1038/srep24361)
Supplement: Supplementary Information [file srep24361-s1.pdf]

Supplementary Discussion

Planktonic events may cause polymictic-dimictic regime shifts in temperate lakes

Tom Shatwell, Rita Adrian, Georgiy Kirillin

Discussion of errors in temperature simulation by one-dimensional lake models

The hydrodynamic model FLake used in this study assumes simplified lake morphology with a flat basin. This enables the use of the self-similarity principle to estimate the temperature profile and drastically increases processing speeds, but may also lead to discrepancies between modelled and measured bottom temperatures. Therefore, we collated the errors from a number of other models to try to assess what would be acceptable here. Perroud, et al. [1] compared and described root mean square errors (RMSE) for different models in simulations of deep Lake Geneva. Here they compared the one-dimensional hydrodynamic models HLM, DYRESM, SIMSTRAT and FLAKE, which produced RMSEs in near-surface water of about 4 °C, 2.5 – 3.5 °C, 2-3 °C, and 3 °C, respectively. The RMSEs at 15-35 m depth for the 4 models were about 7 °C, 3-5 °C, 2-3 °C, and 4 °C, respectively. The errors in deep water temperature (50-300 m) were generally less than 1 °C. In the comparison of Thiery, et al. [2], the centred (and thus slightly lower) RMSEs of 7 models in a meromictic African lake were 0.5-1 °C at 5m depth, 1-2 °C at 30 m depth, and about 2-4 °C at 60 m depth. The model DYRESM has been shown to model surface water and deep water (>30 m) temperature quite accurately after calibration, with mean absolute errors (MAE) typically less than 1 °C in surface and deep water in Lake Kinneret[3] (Israel), or RMSEs between 1 and 3 °C in Lake Ammersee[4] (Germany) and also in Harp Lake[5] (Canada). However, DYRESM does not perform as well in reproducing temperatures in the metalimnion, with higher errors (> 1.5 °C) in these cases at ca. 5 – 20 m depth.

For comparison, we also simulated Lake Heiligensee using the model GLM v2.0.0 with the same forcing data as used in the Flake simulations. Here GLM was configured to the morphology of Heiligensee with approximate hypsographic information and basin dimensions. The simulations were performed uncalibrated with the default parameters and no inflows. The RMSEs for surface temperature (T_s) and bottom temperature (T_b) estimated at 5.5 m depth in these simulations were 3.1 °C and 2.9 °C, respectively, which are slightly greater than the errors with FLake. Thus models with a more complex layer structure do not necessarily perform better than FLake for these intermediate depth lakes.

Therefore it appears that errors less than 2 °C can be expected for surface temperatures and also deep waters in temperate lakes (which maintain relatively constant temperatures near 4 °C). However, models do not seem to reproduce temperatures at intermediate depths as accurately, as is the case with bottom temperatures of marginal lakes (see Introduction in the main text for an explanation), with errors generally greater than 2 °C and also regularly greater than 3 °C. With RMSEs between 2 and 3 °C, the FLake model can thus reasonably reproduce bottom temperatures within acceptable levels of error compared to other models. In our application with a "conceptual" lake, in which we attempted to abstract from responses specific to particular lakes, we think that the level of error is acceptable to draw the conclusions within the stated context.

References

1 Perroud, M., Goyette, S., Martynov, A., Beniston, M. & Anneville, O. Simulation of multiannual thermal profiles in deep Lake Geneva: A comparison of one-dimensional lake models. *Limnol. Oceanogr.* **54**, 1574-1594, doi:DOI 10.4319/lo.2009.54.5.1574 (2009).

2 Thiery, W. *et al.* LakeMIP Kivu: evaluating the representation of a large, deep tropical lake by a set of one-dimensional lake models. *Tellus Ser. A-Dyn. Meteorol. Oceanol.* **66**, doi:10.3402/tellusa.v66.21390 (2014).

3 Gal, G. *et al.* Simulating the thermal dynamics of Lake Kinneret. *Ecol. Model.* **162**, 69-86, doi:10.1016/S0304-3800(02)00380-0 (2003).

4 Weinberger, S. & Vetter, M. Using the hydrodynamic model DYRESM based on results of a regional climate model to estimate water temperature changes at Lake Ammersee. *Ecol. Model.* **244**, 38-48, doi:10.1016/j.ecolmodel.2012.06.016 (2012).

5 Hadley, K. R. *et al.* Climate warming alters thermal stability but not stratification phenology in a small north-temperate lake. *Hydrol Process* **28**, 6309-6319, doi:10.1002/hyp.10120 (2014).
